# Editorial: Insights in neuro-otology: 2021 and 2022

**DOI:** 10.3389/fneur.2022.1104573

**Published:** 2022-12-07

**Authors:** Michael Strupp, Hans Straka, Sun-Young Oh

**Affiliations:** ^1^Department of Neurology and German Center for Vertigo and Balance Disorders, Ludwig-Maximilians-University, Munich, Germany; ^2^Faculty of Biology, Ludwig-Maximilians-University, Planegg, Germany; ^3^Department of Neurology, School of Medicine, Jeonbuk National University, Jeonju, South Korea

**Keywords:** vertigo, dizziness, vestibulo-ocular research, bilateral vestibulopathy, acute unilateral vestibulopathy, nystagmus, Menière's disease, functional dizziness

## Introduction

The Research Topic “*Insights in neuro-otology: 2021 and 2022*” covers, as the title indicates, a broad range of topics from basic research on the vestibular system to clinical studies and trials. It brings together many influential researchers in their specific areas of expertise and presents relevant key findings from the last 2 years. Thereby, it also connects the various levels of research and will stimulate translational and back-translational research. Twenty-seven articles with a total of 126 authors were published in this Research Topic, and so far, it has received more than 22,000 views and has been downloaded more than 3,700 times. This Editorial briefly summarizes the background and the key findings and conclusions of the various contributions, providing an overview that will hopefully prompt the reader to read these articles in full.

## Basic and applied vestibular research

The connection between mammalian type I hair cells and calyx afferents forms a highly specialized synapse that is excellently suited for the transmission of high-frequency, high-acceleration head motion profiles. Contini et al. coherently describe, in a mini review, the multiple communication modes of the calyx synapse. The conventional glutamatergic quantal transmission on the millisecond timescale by these synapses is assisted by low-pass filtered transduction currents mediated by potassium ions that accumulate in the synaptic cleft. This mechanism depolarizes hair cells and afferents and thereby enhances the conventional transmission, both pre- and post-synaptically. A third form of transmission in the microsecond range exploits the large voltage- and ion-sensitive cleft-facing conductances of the hair cells and the calyx, adding collectively to the large range of response dynamics of irregular firing vestibular afferent fibers.

Cultures of isolated spiral ganglion cells usually contain cell bodies of auditory afferent fibers but also cochlear fibroblasts, which, in the case of cochlear implants, can produce undesired fibrous tissue around the electrode. Anacker et al. succeeded in purifying fibroblasts following removal of neurons, and by increasing the ratio of fibroblast to glial cells, verified by differential staining for vimentin, S100B-protein, and 200-kD neurofilament. Using Thy1-glycoprotein and p75NGFR immunolabeling allowed fluorescence-based cell sorting, providing an almost pure cochlear fibroblast cell culture. These cells exhibit a fibroblast-specific morphology, are stable for multiple proliferation rounds, and robustly survive cryopreservation. This offers the possibility of conducting *in vitro* studies to find possible conceptual solutions for minimizing fibrous scar formation.

In their study with 25 healthy participants, Tarnutzer et al. demonstrate that heading direction is significantly biased by preceding short-term whole-body roll orientation while lying horizontally. This finding underlines the broad impact of a shifting internal estimate of the direction of gravity. The authors conclude that, in a broader context, the observed bias in perceived straight ahead emphasizes that getting up in the morning is a vulnerable period with an increased risk of falls and fall-related injuries due to an inability of optimally tuned internal estimates of the direction of gravity and the direction of straight ahead. This article therefore has a direct impact and helps to explain the high risk of falls after a good night's sleep.

Many animal and clinical studies have extensively reported prominent and long-lasting spatial cognitive deficits in bilateral vestibulopathy (BVP), along with some evidence of hippocampal atrophy. Nguyen, Nam et al. report that these impairments were ameliorated by application of five consecutive daily sessions of noisy galvanic vestibular stimulation (nGVS), as evidenced by the substantial improvement of poor behavioral performance during the Y-maze and MWM tasks in mice with a surgically incomplete BVP. This weak galvanic current, which acts at the sensory endorgans and the spike trigger zone of primary vestibular afferents (inhibitory anode and excitatory cathode that can be amplified by the “stochastic resonance” phenomenon) and assists in rebalancing bilateral vestibular inputs, has recently shown positive effects in improving spatial cognition as well as postural and locomotion deficits in patients with BVP with residual function.

Vestibulo-ocular reflexes (VOR) stabilize retinal images by counter-rotating the eyes during head movements, ideally with a unity gain. However, in many species, and in elderly humans or patients with a vestibular impairment, the VOR gain is far from compensatory. Glasauer and Straka suggest in their study that such a suboptimal VOR gain reflects an adaptation to the sensory and motor signal variability. Since sensorimotor noise can disturb stabilizing eye movements, minimization of the overall retinal image slip should incorporate the effects of both noise components and the dynamic constraints of the signal processing. This premise was confirmed in *Xenopus laevis*, where computational calculations correspond to the observed variability of eye movements and the overall low VOR gain. This thus indicates that lower VOR gains in elderly humans or recovered vestibular patients may be an optimal adaptation given higher noise levels rather than a direct consequence of the principal damage.

Vestibular function is clinically evaluated by the video head impulse test combined with video-oculography (VOG), even though the latter method is occasionally challenging due to pupil detection issues. This prompted Pleshkov et al. to compare VOG with electro-oculography (EOG) as a technically more robust method for eye motion quantification. Horizontal eye movements in healthy human subjects were recorded with VOG and EOG during bidirectional head impulses and recorded magnitudes were cross-correlated and statistically evaluated. Eye movements obtained with either method correlated well with each other. However, EOG recordings were symmetric for bidirectional head impulses, while VOG recordings yielded a clear left-right asymmetry. This outcome suggests that EOGs are well-suited for eye movement recordings and might in fact represent a more objective technology.

A so far unknown positional preference was evaluated by Park et al. during the acute phase of an acute unilateral vestibulopathy by assessing the severity of vertigo with a visual analog scale (VAS). Patients were characterized with VOG, including standard tests such as spontaneous nystagmus during sitting, head rolling to both sides or ocular and cervical vestibular-evoked myogenic potentials. About a third of the patients complained of more severe vertigo during lying on the affected side compared to the healthy side with (36%) or without (30%) visual fixation. Both groups had significantly higher VAS and maximal slow phase velocities of the spontaneous nystagmus in all positions except lying on the impaired side, compared to those without positional preference. In the absence of other differences between the groups, the positional preference might reflect damage to the activation of the tonic but not the phasic otolithic system.

Head-shaking nystagmus can derive from asymmetric peripheral vestibular signals or from lesions, mostly in the cerebellum, that affect the velocity storage. In a case study, Filippopulos et al. report on a patient with recurrent episodes of vertigo, induced by fast head movements and accompanied by a severe head-shaking nystagmus with a long time constant. In the absence of other criteria for vestibular/auditory or neurological disorders, the authors suggest the presence of a clinical entity termed “acquired idiopathic head shaking nystagmus” as a rare cause of episodic vertigo with a distinct pathology of the cerebellar velocity storage mechanism that becomes apparent during fast head movements.

Based on the similarity of the recurrent spontaneous vertigo and absence of other pathological signs, Lee and Kim in a commentary suggest that this patient suffers from a similar pathology as a previously described group of patients. Based on the characterization of these latter patients, the authors suggest that an unstable, asymmetric velocity-storage mechanism is the common pathological mechanism for the benign recurrent vertigo with head-shaking nystagmus and for acquired idiopathic head-shaking nystagmus.

Vestibular dysfunction can be detected by observing corrective saccades/re-fixation saccades that return the eyes to the target during a head impulse test. Corrective saccades are defined as covert or overt depending on whether the onset occurs before or after the end of the head impulse, respectively. In the absence of a clear mechanistic origin, Iwasaki et al. examined the role of neck proprioceptive signals as a trigger for these saccades. In patients with vestibular dysfunction, head and eye movements were recorded during the head or body impulse test, where during the latter maneuver, the head is fixed to the body. While the number of corrective covert saccades was similar in both conditions, there were significantly fewer overt saccades during the body impulse test. This result suggests that neck somatosensory inputs contribute to the generation of overt saccades, thereby reinforcing the VOR during high-frequency head movements.

## Clinical vestibular research

There have already been many publications on vestibulo-cochlear manifestations in COVID-19 cases with contradicting findings, but one must always consider that there were vestibular disorders even before this pandemic. Kaliyappan et al. critically review basic mechanisms that may lead to an impairment of vestibular and audiological function by SARS-CoV-2, namely the role of the angiotensin-converting enzyme 2 receptor in various parts of the brain. However, critically viewing the many case reports and new case series published so far, there is currently no evidence for a major impact of SARS-CoV-2 on either the peripheral vestibular or the auditory system, which is also true for the vaccines against SARS-CoV-2. There is still a great need for clinically relevant and validated scales to quantify the impact of vestibular disorders on functionality and quality of life.

In their article, Kristiansen et al. describe the variations in signs and symptoms in 107 patients with persistent dizziness using physical tests and self-reported outcomes across three severity levels of the Dizziness Handicap Inventory (DHI). Participants performed physical tests, psychological measures and various scales for the quality of life. There was a trend toward worse scores on physical tests and self-reported measurements with increasing DHI severity level. However, several signs and symptoms may not be detected by the DHI and therefore the authors recommend a combination of outcomes in patients with persistent dizziness.

Another article in this Research Topic focuses on a similar topic: development and content validity of the bilateral vestibulopathy (BVP) questionnaire by van Stiphout, Hossein et al. The aim was to measure the burden and severity of the full spectrum of bilateral vestibulopathy symptoms and to assess its impact on daily life. A two-step approach was used: (1) initial item generation and (2) phase and content validity testing. This questionnaire consists of two sections: (1) 50 items scored on a six-point Likert scale, e.g., on imbalance and cognitive symptoms; and (2) four items on a visual analog scale from 0 to 100 on limitations in daily life, perceived health and expectations regarding recovery. The authors conclude that this questionnaire can be used to characterize current self-reported symptoms and disability and the burden of disease and as an outcome measure in clinical practice and clinical studies.

Another article by van Stiphout, Pleshkov et al. also focuses on BVP, namely the patterns of vestibular impairment and its relationship to etiology. A total of 173 subjects from three tertiary referral centers in Europe with the diagnosis of BVP according to the Barany Society diagnostic criteria were included. They underwent a full diagnostic workup. The major findings were as follows: vHIT and caloric testing seem to be more sensitive for measuring vestibular impairment, whereas the Torsion Swing Test is more suited to measuring residual vestibular function. No striking patterns of vestibular impairment in relation to etiology were found. Finally, the authors advised that one should carefully examine every patient for its overall pattern of vestibular impairment in order to make well-informed and personalized therapeutic decisions, i.e., if vestibular implants are considered in the future.

There are currently three types of stimulation for vestibular evoked myogenic potentials: air-conducted sound, bone-conducted vibration and galvanic vestibular stimulation. For bone-conducted vibration, various bone vibrators have been used, e.g., the mini-shaker and the RadioEar. In a methodologically important study, Zhang et al. evaluated normal values and the effect of aging on bone vibrator-induced cervical and ocular vestibular evoked myogenic potentials (cVEMPs and oVEMPs). With aging, the cVEMP response amplitude decreased, while the threshold and the latency increased. The same was true for oVEMPs. This shows the importance of each laboratory with a sufficient number of patients ideally generating its own reference values in healthy individuals for different age groups.

Menière's disease is evidently the result of various underlying etiologies leading to such a phenotype. In addition to genetic factors, inflammation is one of the underlying etiological factors. Using transcriptome analysis, Choi et al. compared 39 patients with Menière's disease with 39 controls using peripheral blood monocular cells to evaluate differentially expressed genes. Four hundred and fifteen genes were upregulated and 581 were downregulated in the Menière's disease group. The downregulated genes were mainly associated with the immune system pathways, including the major histocompatibility complex, which may contribute to the development of Meniere's disease and may have implications for a targeted treatment in the future based on the genetic profile of each patient: precision medicine.

Using the “HYDROPS” (HYbriD of the reversed image of positive endolymph signal and native image of positive perilymph signal) imaging magnetic resonance (MR) protocol, Han et al. describe a significant positive correlation between some clinical and laboratory parameters in Menière's disease (MD), including the diagnostic scale of MD (possible, probable, and definite), pure tone average, low tone average, and canal paresis, but no such correlation with disease duration. These findings suggest that MR-based radiological evaluation of endolymphatic hydrops (EH) is useful for determining the extent and severity of MD, as well as the pathophysiological relationship between Menière's disease and EH.

In a case series, Gianoli et al. describe a new form of labyrinthine dehiscence: horizontal semicircular canal dehiscence at the tympanic segment of the facial nerve in 16 patients. The tympanic segment of the facial nerve crosses over the ampullated end of the horizontal semicircular canal. This type could also be the source of persistent symptoms after regular superior semicircular canal dehiscence surgery.

In a case report, Hofmeyr et al. describe the precise diagnosis of petrous apex IgG4-related disease by middle cranial fossa craniotomy and temporal bone biopsy in a patient with diplopia and unilateral headache, which had major therapeutic consequences because, after a precise histological diagnosis, the patient was treated with steroids, which led to a complete recovery.

Given the recent evidence that high-intensity galvanic stimulation (GVS) current can cause spatial cognitive performance errors, GVS threshold determination is required to optimize intervention. Nguyen, Kang et al. show that in a simultaneous analysis of the three impacts of vestibular and cutaneous perception and oculomotor response, induced by direct current galvanic vestibular stimulation (DC-GVS) with surface electrodes placed at the bilateral mastoid in healthy subjects, the mean vestibular threshold was lower than the mean oculomotor threshold but similar to the mean cutaneous threshold. This vestibular threshold was influenced by gender characteristics (significantly higher in males than in females) but not by the GVS paradigm (cathode right anode left vs. cathode left anode right).

In a review of studies published over the last 2 years exploring the contribution of the vestibular system to motor and cognitive processes, Smith provides insight into the interactions of vestibular information and hippocampal and striatal functions. Recent findings of vestibular impairments in Parkinson's disease and Alzheimer's disease have important implications for a comprehensive understanding and optimal treatment of these disorders.

In a model to evaluate the influence of individual sensory impairments, Lucas et al. discovered that olfactory dysfunction, gait impairment, and sensorineural hearing loss were significantly associated with increased risk of cognitive impairment, with olfactory dysfunction being the strongest predictor of cognitive impairment. Subjects with dysfunction in all three domains had a higher risk of cognitive impairment than those with impairment in fewer than two domains, indicating that multisensory dysfunction may contribute synergistically to cognitive impairment. These findings support the hypothesis that cognitive impairment and dementia are associated with sensory impairments in a variety of domains, common in elderly subjects, and early therapeutic intervention potentially prevents cognitive decline.

Oh et al. show that injection of botulinum toxin type A (BTX-A) in patients with vestibular migraine (VM) resistant to conventional prophylactic therapies significantly reduces the frequency of migraine and vertigo episodes and improves the Headache Impact Test-6 (HIT-6) score, Migraine Disability Assessment (MIDAS), Dizziness Handicap Inventory (DHI), Vertigo Symptom Scale (VSS), and anxiety and depression scores 2 months after treatment. Analysis of resting-state functional MRI data also revealed an increased functional connectivity of the right superior temporal gyrus, left post-central gyrus, right supramarginal gyrus, and right middle temporal gyrus, all of which were negatively correlated with the MIDAS score. This suggests that BTX-A may enhance intrinsic vestibular, nociceptive, and cross-modal interactions within the multisensory cortical network and is effective at improving vertigo and migraine attacks in VM.

De Vestel et al. conducted a cross-sectional study to compare clinical balance and visual dependence tests among persistent postural-perceptual dizziness (PPPD) patients, dizzy non-PPPD patients, and healthy subjects (HS) to determine whether these clinical tests can help to identify PPPD. PPPD patients were more visually dependent with significantly higher Visual Vertigo Analog Scale (VVAS) scores but did not have a worse postural balance compared to dizzy non-PPPD patients. This suggests that the VVAS had the highest discriminative value for identifying PPPD in chronic dizziness.

For the treatment of PPPD, Im et al. report that active transcranial direct current stimulation (tDCS) was not significantly more effective than sham tDCS for alleviating dizziness symptoms in a randomized, double-blind, sham-controlled trial of tDCS. The applied tDCS (20 min per session, 15 sessions over 3 weeks), targeting the dorsolateral prefrontal cortex, did not achieve significant symptom reduction, indicating the need for further research in PPPD using different stimulation protocols of tDCS with respect to stimulation site or number of sessions.

## Author contributions

All authors listed have made a substantial, direct, and intellectual contribution to the work and approved it for publication.

